# Recent tropical cyclone changes inferred from ocean surface temperature cold wakes

**DOI:** 10.1038/s41598-021-01612-9

**Published:** 2021-11-15

**Authors:** Shuai Wang, Ralf Toumi

**Affiliations:** Department of Physics, Imperial College London, London, SW7 2AZ UK

**Keywords:** Natural hazards, Atmospheric science

## Abstract

It has been challenging to detect trends of tropical cyclone (TC) properties due to temporal heterogeneities and short duration of the direct observations. TCs impact the ocean surface temperature by creating cold wakes as a “fingerprint”. Here we infer changes of the lifetime maximum intensity (LMI), size and integrated kinetic energy from the cold wakes for the period 1982–2019. We find a globally enhanced local cold wake amplitude 3 days after the LMI of − 0.12 ± 0.04 °C per decade whereas the cold wake size does not show any significant change. Multivariate regression models based on the observed ocean cooling, the TC translation speed and the ocean mixed layer depth are applied to infer LMI and TC size. The inferred annual mean global LMI has increased by 1.0 ± 0.7 m s^−1^ per decade. This inferred trend is between that found for two directly observed data sets. However, the TC size and the TC destructive potential measured by the integrated kinetic energy, have not altered significantly. This analysis provides new independent and indirect evidence of recent TC LMI increases, but a stable size and integrated kinetic energy.

## Introduction

Tropical cyclone (TC) intensity and size are two main properties affecting the TC destructive potential^1^. TC intensity is conventionally measured by the sustained maximum wind speed near surface. TC intensity is limited by TC potential intensity^2,3^ and the lifetime maximum intensity (LMI) is closest to this upper limit in a TC life cycle. TC thermodynamics demonstrates a strong dependence of TC intensity on sea surface temperature (SST) via enthalpy up-take at sea surface^4^. From a theorical perspective it is expected that a warmed ocean due to greenhouse gas emissions will increase the mean state of TC potential intensity^5,6^. It may consequently shift the LMI distribution towards greater intensities in a more favorable environment.

This prediction of long-term TC intensity increase has been examined by TC observations. The “best-track” data contains the most comprehensive TC observations in the past 150 years^7^. The best track records are considered to be globally complete only since 1982 when all ocean basins with TC activities were routinely monitored by satellites^8^. It is found with the best track that the annual mean LMI of global TCs (LMI at least category 1) increases significantly by 2.0 m s^−1^ per decade for 1982–2009^8^, and 1.5 m s^−1^ per decade for 1982–2019^9^ for major TCs (LMI at least category 3), respectively. The proportion of the major TCs has also been found increasing significantly for 1979–2017^10^.

Best tracks are the most comprehensive TC observation data base. The best-track maximum wind speed largely depends on a cloud-pattern-based index, and the maximum wind speed is then determined from that index and a conversion table that only provides discrete intensities. Therefore, any statistically significant intensity trend less than the intensity change interval may not be robust. Best tracks have been collected for decades with the best observational techniques and analysis protocols of the time. However, this naturally creates temporal heterogeneities and may lead to unphysical detections, for example, the underestimation of TC frequency in the Atlantic prior to the satellite era^11^, and the intensity discontinuity in the western North Pacific due to the termination of aircraft reconnaissance^12^.

To partly resolve this issue, globally and temporally consistent TC records, ADT-HURSAT^8,10^, are developed based on satellite observations and the advanced Dvorak technique^13^. The ADT-HURSAT sacrifices some valuable flight observations for homogeneity but arguably allows more robust trend detection of TC intensity. It is found with the ADT-HURSAT that the positive trend of annual mean LMI of global TCs is not statistically significant for 1982–2009^8^, which is contradictory to the significant trend found with the best track. This discrepancy of the statistical significance of LMI trends makes it difficult to answer if anthropogenic effect has changed the TC intensity, or the significant positive trend of TC intensity is just due to technology change, rather than nature or anthropogenic climate changes. More evidence for the long-term change of TC intensity is needed.

Compared to the TC intensity, TC size has a much shorter global observation in the best track, which is only available since the twenty-first century^7^. The TC size can be measured by the radius of, e.g., maximum wind speed or the gale-force wind speed. A significant shrinking of the radius of gale-force wind (R_18_) was found for global TCs in the best track^14^. However, no significant long-term trends of TC outer size was reported based on the inferred imagery for 1978–2011^15^. The Integrated Kinetic Energy (IKE) has been shown to be a superior metric to indicate the overall TC destructive potential^16^. The IKE is controlled by both TC intensity and size^17^. The temporal heterogeneities of TC intensity observations and a lack of TC size records make it challenging to examine the long-term trend of TC IKE. An analysis of global climate models shows no significant change of TC IKE in the future under climate change^18^. However, more long-term historical change of TC IKE is unknown, and we seek to fill this gap here.

In this study we will provide a new evidence of TC intensity, outer size, and destructive potential changes from an indirect angle, that is, by inferring these TC trends from the observed TC impact on sea surface temperatures. TC properties are constrained by ambient environmental conditions. Reversely the environment also has a memory of TC passages. One example is TC-induced SST cooling or cold wake^19^, the amplitude of which is modulated primarily by three factors: the TC intensity^20^, translation speed^21^ and upper ocean thermal structure^22^. A cooling of the ocean wakes caused by TCs has been recently reported^23^, which is found to be spatially correlated with TC intensity changes, but this observed change has not been used to infer the TC intensity trend. It has also been shown that the size of cold wakes and TC size can be related^24^. Here we will establish the statistical relationship between the SST cooling amplitude/size, TC intensity/size, and environmental conditions at LMI via multivariate linear regression, and then estimate the TC intensity/size and thus the IKE trends with observed changes of oceanic footprints due to TC passages.

## Results

**Figure 1:**
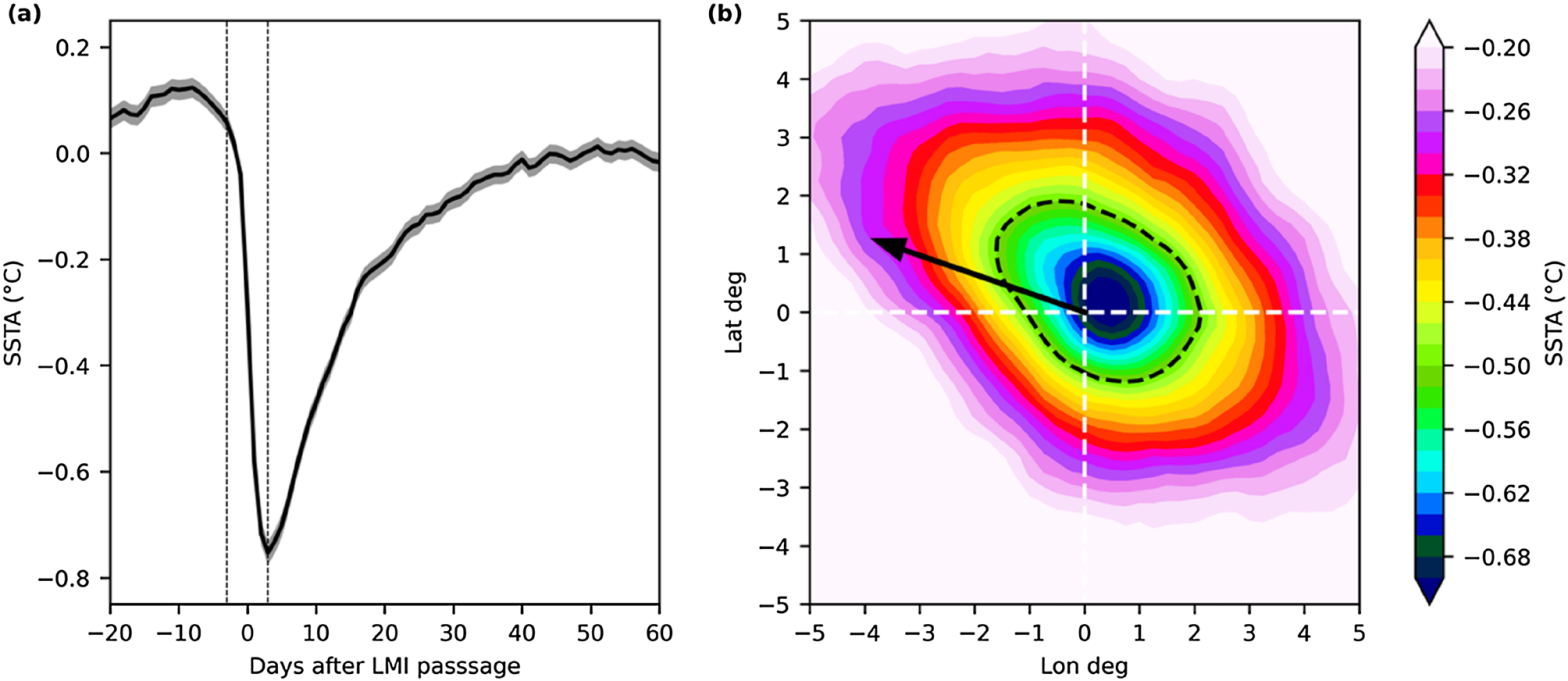
Composite of TC-induced SSTA for 1982–2019. (**a**) Mean time series of SSTA at the location of LMI. The shading shows one standard error. The thin dash black lines highlight 3 days before and after LMI. (**b**) Mean SSTA map 3 days after LMI. The location of LMI is at the origin. The black arrow shows the mean translation direction (not magnitude). The black dash line shows the area of less than − 0.5 °C SSTA.

Figure 1a shows the mean time series of SST anomaly (SSTA, see "Methods" section) at the location of LMI of all global TCs with LMI ≥ 33 m s^−1^ for 1982–2019. The ocean surface starts to be cooled down at a relatively faster rate 3 days before the LMI passage. The mean maximum cooling (i.e., minimum SSTA) is observed 3 days after the LMI passage. We therefore opt to use the difference of SSTA 3 days after and before LMI to define the TC-induced ocean cooling amplitude, i.e., ΔSSTA.

Figure 1b shows the composite of SSTA map 3 days after the LMI passage. We find stronger ocean cooling along the TC moving direction with a slight shift to the right of the motion. The general cooling pattern appears to be in a circle shape, which allows us to calculate the cooling size in terms of the radius of cooling from the TC center, i.e., R_C_, by assuming a circle cooling pattern with an equivalent area of the actual cooling region. The mean R_18_ of the selected TCs in this study is 206 km. We find a close match of R_C_ (216 km) to R_18_ if − 0.5 °C is chosen for the definition of TC-induced cooling area boundary (the black dash line in Fig. 1b). Thus, in the following analysis “ − 0.5 °C” is chosen as a threshold for the calculation of R_C_.

**Figure 2:**
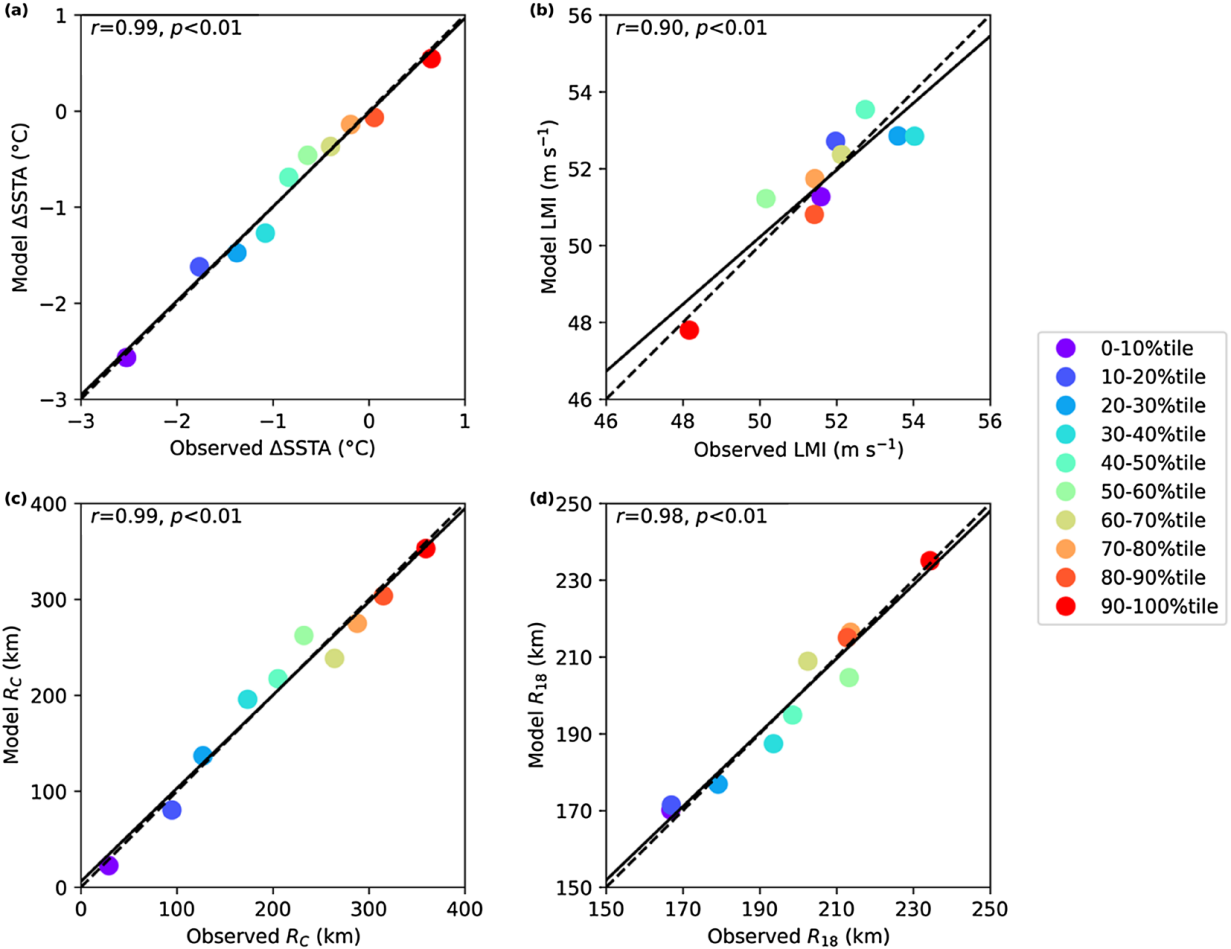
Multivariate linear regression after binning global TCs into ten groups based on the deciles of TC-induced (**a**, **b**) sea surface cooling amplitude (ΔSSTA), and (**c**, **d**) sea surface cooling size (R_C_). The dots change from cold color for strong ΔSSTA or small R_C_, to warm color for weak ΔSSTA or large R_C_. The mean ΔSSTA, LMI, C and MLD in each bin are used for the regression in (**a**, **b**), and the mean R_C_, R_18_, C and MLD are used for the regression in (**c**, **d**). The regression model is Eq. (1) in (**a**), Eq. (2) in (**b**), Eq. (3) in (**c**) and Eq. (4) in (**d**). The Pearson correlation coefficient (*r*) and the *p*-value between the model and observed properties are given in the legends. The solid line shows the best linear fit and the dashed line shows y = x.

We next establish the statistical relationship between ΔSSTA and LMI, TC translation speed around LMI (C, see "Methods" section) and the mixed layer depth at LMI (MLD, see "Methods" section) by grouping the TCs according to the deciles of the cooling, ΔSSTA (Fig. 2a, b). The multivariate linear regression shows an excellent representation of grouped ΔSSTA (Fig. 2a), with a regression model of

1 $$ \Delta SSTA = - 0.2LMI + 1.0C + 0.1MLD $$

where all three regression coefficients are significant at 5% level. A very similar regression result can be obtained by eliminating the MLD term globally (*r* = 0.97, *p* < 0.01) and in individual basins. However, the regression performs much less well if only the global LMI is used as a single predictor for ΔSSTA (*r* = 0.53, *p* < 0.01).

We next rearrange the statistical relation in Eq. (1) by using grouped ΔSSTA, C and MLD as explanatory variable to regress on grouped LMI. Figure 2b shows a good representation of grouped LMI (*r* = 0.90, *p* < 0.01), with a regression model of

2 $$ LMI = - 5.7\Delta SSTA + 5.7C + 0.6MLD $$

where like Eq. (1), all the coefficients are significant at 5% level.

A similar analysis is then conducted between R_C_ and R_18_, C and MLD by grouping the TCs based on the deciles of R_C_ (Fig. 2c, d). An excellent representation of grouped R_C_ is found with a regression model of

3 $$ R_{C} = 3.6R_{18} - 33.6C - 11.3MLD. $$

If we rearrange the relationship in Eq. (3) to represent grouped R_18_ with R_C_, C and MLD, the multivariate linear regression again gives an excellent representation (Fig. 2d), and the regression model is estimated as

4 $$ R_{18} = 0.3R_{C} + 9.1C + 3.2MLD. $$

Similar to Eqs. (1) and (2), all the fitted coefficients are significant at 5% level in Eqs. (3) and (4).

Equations (2) [or (4)] provides a way of inferring LMI [or R_18_] from observed ΔSSTA [or R_C_], C and MLD. We next conduct such an estimation for LMI and R_18_, respectively, with the observations of individual TCs. Since the regression models [Eqs. (2) and (4)] are established with grouped quantities, we do not expect a good match between estimated and observed LMI and R_18_ of individual TCs. The individual LMI and R_18_ will be controlled by many other more important factors than the ocean cooling. However, the maximum ocean cooling amplitude and size themselves are direct responses to the TC intensity and size. Here we try to use the long-term changes of ΔSSTA [or R_C_], C and MLD to represent LMI [or R_18_] with the statistical relationship as shown in Eqs. (2) [or (4)].

**Figure 3:**
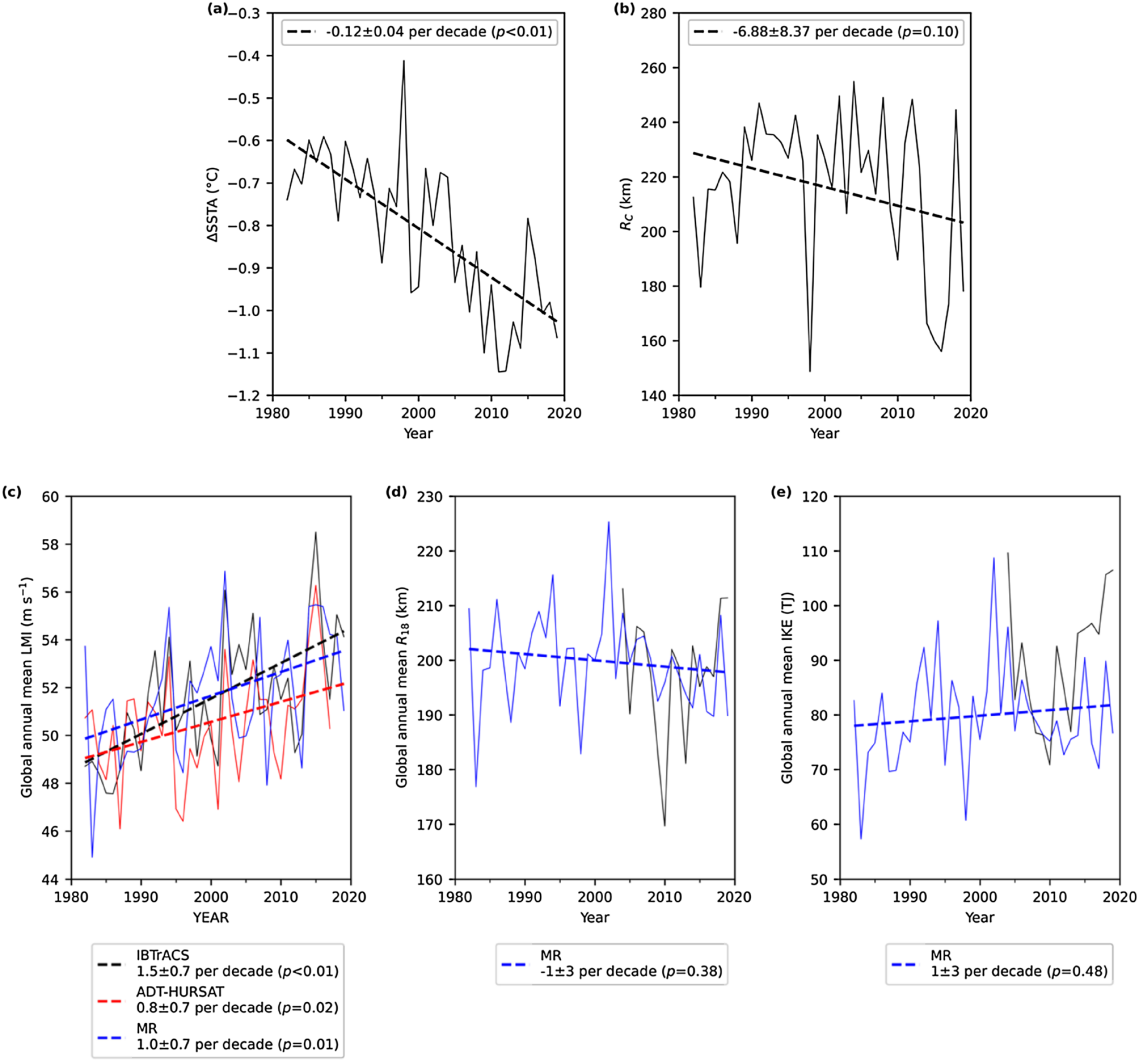
Annual mean time series of global TCs. (**a**) TC-induced ΔSSTA measured 3 days after the LMI passage at the location of LMI relative to the values 3 days before. (**b**) TC-induced R_C_ measured 3 days after the LMI passage. (**c**) Observed and inferred (with multivariate regression, MR) annual mean LMI. The observed annual trends are extracted from the IBTrACS and ADT-HURSAT data sets for TCs (LMI ≥ 33 m s^−1^). (**d**) Observed and inferred R_18_ at LMI. (**e**) Observed and inferred IKE at LMI. The mean ± 95% confidence interval of the linear trend (thick dash line) is given in the legend with the *p*-value.

Figure 3 shows the trend analysis of ocean cooling and inferred TC properties. A significantly larger ocean cooling amplitude in recent decades is found at a rate of − 0.12 ± 0.04 °C per decade (ΔSSTA, Fig. 3a). However, the ocean cooling size (R_C_, Fig. 3b), TC translation speed (C, Fig. S1a) and upper ocean stratification (MLD, Fig. S1b) do not show any significant change.

Figure 3c shows the annual mean trend of inferred LMI with Eq. (2) compared to the observations from the best tracks and ADT-HURSAT. The inferred annual mean LMI is significantly correlated with best track and ADT-HURSAT time series after detrending (*r* = 0.35 and 0.37, *p* = 0.03 and 0.02, respectively). All three trends are statistically significant but with different mean trends. The strongest trend (1.5 ± 0.7 m s^−1^ per decade) is found in the best track, whereas the ADT-HURST trend (0.8 ± 0.7 m s^−1^ per decade) is only about half of the mean best-track trend. Our inferred mean LMI trend (1.0 ± 0.7 m s^−1^ per decade) lies between the two observations and shows the same trend confidence interval.

Figure 3d shows the annual mean trend of inferred R_18_ with Eq. (4) compared to the observations in the best track. There is also a significant correlation between the two detrended annual mean time series after 2004 (*r* = 0.54, *p* = 0.03). The inter-annual correlation is higher than that found for the inferred intensity. However, different from the three intensity trends, there is no significant trend of the inferred R_18_.

With the observed LMI and inferred R_18_ we next calculate the TC integrated kinetic energy for 1982–2019 (Fig. 3e, see "Methods" section for the calculation of IKE). The inferred and observed IKEs show a surprisingly high correlation after detrending post 2004 (*r* = 0.70, *p* < 0.01), which gives us high confidence. Even though the LMI increases significantly for 1982–2019, the global annual mean TC IKE is stable at around 80 TJ at LMI without any significant long-term change. A consistent lack of significant trend of IKE is also found in Fig. S2 by applying an alternative wind profile model^25^. Furthermore, the results in Fig. 3 can be qualitatively reproduced by replacing the equivalent cooling radius, R_C_, with the cooling area (Fig. S3). The lack of IKE trend is consistent with the insignificant R_18_ trend, which emphasizes the important role of TC size in the overall TC destructive potential change.

## Discussion

In this study, we show that the ocean surface cooling can be used as an independent measure of long-term global changes of TC intensity, size and destructive potential. Given the uncertainty of the best track data, this new analysis can provide extra evidence of TC feature changes in the past decades. The established statistical relationship between ΔSSTA, LMI, C and MLD correctly reflect the known major factors on TC-induced ocean cooling. This shows the ability of the multivariate regression model for TC trend detection. However, this does not mean that the regression model based solely on ocean conditions can be used for a case-by-case prediction tool as the TC features are largely modulated by atmospheric conditions^26^. The ocean cooling is a “fingerprint” of the TC not a predictor of the TC.

Equation (1) suggests that a more intense TC, a slower translation and/or a shallower warm water layer can lead to a stronger TC-induced ocean cooling. This is as expected and in line with many studies^24,27–32^. Our regression analysis also suggests that the ocean cooling can be largely explained by a linear superposition of the effects of TC intensity and translation speed whereas the MLD plays a much weaker role, which agrees with previous findings^27^. The regression model can perform equally well without the MLD term. Although we use monthly MLD observation in the analysis, we would not expect to see qualitive changes of the results if daily MLD observation at the location prior to the LMI were available. Spatial migration of TC activities has been observed in the past four decades^33–35^. However, any potential TC-related changes of MLD associated with these spatial migrations, based on our analysis, may not change the statistical relationship between TC intensity and ocean cooling.

Our result in Fig. 2b suggests a weaker sensitivity of ocean cooling to TC intensity when the LMI is more than 50 m s^−1^. This reduced sensitivity agrees with the previously found leveling-off of ocean cooling with increase TC intensity^36^. Even though, by including the effect of TC translation, the grouped LMI can still be correctly obtained with the regression model.

The observed sea surface cooling trend is larger than the significant digits of sea surface temperature obtained by observation. However, the observed sea surface cooling observation may also experience temporal heterogeneities due to the improvement of observational techniques, which is a similar issue as the best track has. Our finding of the enhance TC-induced sea surface cooling is in line with a recent study reporting a cooling trend of TC wakes by about 0.05 °C per decade^23^, which was also validated with the microwave satellite SST data set. This cooling trend is about half of what we find here, but all TC positions were considered, not just at the LMI as here. As the cooling increases with TC intensity and the LMI trend is more than double the mean intensity trend, our larger cooling trends are understandable. This study makes the first attempt to infer the wind speed trend, but the previous study^23^ did show a high spatial correlation of the cooling enhancement and TC intensity increase.

We find that the cold wake size is dependent on the TC size, which is in agreement with previous findings^24^. We use the ocean response to also infer no recent change in TC size. Compared to the TC intensity studies, observations and projections of TC size trend are scarcer and more uncertain. Some model simulations detected an increase in TC size in a future climate^37–39^. However, a negative trend of R_18_ was also reported in the best track for 2001–2014^14^, but that period may not be long enough for long-term trend detection. Here we provide a new evidence of TC size variation that shows no significant change for 1982–2019, which is consistent with model simulations^40–42^. Due to this lack of significant change in TC size, TC IKE on average remains the same level, which indicates a stabilization of TC destructive potential at LMI, even though the LMI has increased significant during this period. This stabilization, due to the large dependence of IKE on TC size^16,17,43^, would seem to support the climate model simulations which show projected increase in TC intensity but no change in TC integrated kinetic energy^18^. Interestingly, a recent study^9^ also reported no recent change in the acumulated cyclone energy of major TCs. It would thus appear that at least those two integrated tropical cylone wind measures are more stable than the maximum surface wind speed.

## Methods

### Data

The best track are taken from the International Best Track Archive for Climate Stewardship^7^ (IBTrACS, Version 4.0). We only take the records at the standard observational times: 00, 06, 12, 18 Coordinated Universal Time (UTC). A TC may reach the same LMI more than once and if this happens, we only take the first LMI in a TC life cycle. This study includes all global TCs with LMI ≥ 33 m s^−1^ for 1982–2019. Six ocean basins are considered, which are the western North Pacific (WP), eastern North Pacific (EP), North Atlantic (NA), North Indian Ocean (NI), South Indian Ocean (SI) and South Pacific (SP). The best track in the WP, NI, SI and SP are taken from the Joint Typhoon Warning Center, whereas the best track from the National Hurricane Center (also known as HURSAT) is used in the NA and EP. The translation speed (C) is calculated with the best track in a 12-h period centered on the time of LMI.

We use the $$\raise.5ex\hbox{$\scriptstyle 1$}\kern-.1em/ \kern-.15em\lower.25ex\hbox{$\scriptstyle 4$}^{ \circ }$$ daily Optimum Interpolation SST v2.1^44,45^ to extract TC-induced ocean cooling for 1982–2019. The upper ocean thermal structure is characterized by the mixed layer depth (MLD). Due to a lack of high-temporal resolution data we use the 1° monthly mean MLD climatology^46^ developed with Argo profiles to represent the upper ocean thermal condition in the month and at the location of LMIs.

### Ocean cooling

TC-induced ocean cooling is calculated for each TC at the location of LMI from 20 days before to 60 days after the LMI passage. The ocean cooling on each day is estimated as the SST anomaly (SSTA) relative to the mean SST climatology on that day for 1982–2019 at the location of LMI after any linear trend is removed. The amplitude of TC-induced ocean cooling (ΔSSTA) is calculated as the SSTA decrease between 3 days after and before the LMI passage. The TC-induced ocean cooling size (R_C_) is calculated as the radius of the cooling area showing an SSTA less than − 0.5 °C by assuming a perfectly circle cooling patten.

### Multivariate linear regression

We use the LMI to represent TC intensity. For the LMI-related calculation, we use a multivariate linear regression to establish the statistical relationship between ocean cooling amplitude (ΔSSTA), and its three factors: TC intensity (LMI), TC translation speed (C), and upper ocean thermal feature (MLD). Firstly, the complete time series of ΔSSTA, LMI, C and MLD including all TCs are detrended. To reduce the noise level, we then classify all the TCs into the deciles of ocean cooling amplitude. The mean of ΔSST, LMI, C and MLD in each group is used for the multivariate regression analysis.

We use the radius of gale-force wind (18 m s^−1^) at LMI (R_18_) to represent TC outer size. For the R_18_-related regression, the same multivariate linear model is applied as for the LMI, but the ΔSSTA and LMI are related by the R_C_ and R_18_, respectively.

### IKE calculation

The IKE is defined as the sum of kinetic energy at 10 m within R_18_^17^. The IKE calculation requires a complete wind profile within R_18_. An analytic wind profile model^47^ is applied to reconstruct the surface wind distribution. The maximum wind speed, the latitude of TC center and R_18_ are used as three inputs of the analytic wind profile model. This procedure for wind field reconstruction has been fully validated^43^ and applied with global climate model simulations^18^. To examine the robustness of IKE calculation, we also apply an alternative wind profile model—the updated Holland model^25^ that also requires the radius of maximum wind which is obtained from the IBTrACS.

### Statistical significance

The statistical significance in this study is defined at the 5% level. In Fig. 3 we calculate the 95% confidence intervals for the linear trend with the weighted least-squares regression based on the TC count in each year. The standard error of the fit and degrees of freedom are used to generate the confidence bounds for the trend. Any autocorrelation of the time series are examined with the Durbin–Watson test for AR(1). The confidence intervals are adjusted with an updated degrees of freedom if an AR(1) process is detected^48^.

## Supplementary Information

Supplementary Figures.
