# Supplementary material for: Recent tropical cyclone changes inferred from ocean surface temperature cold wakes
Source: Sci Rep. 2021 Nov 15;11:22269. doi: 10.1038/s41598-021-01612-9 (PMC8592988; doi:10.1038/s41598-021-01612-9)
Supplement: Supplementary file 1 — Supplementary Figures. [file 41598_2021_1612_MOESM1_ESM.docx]

Supplementary information for

**Recent tropical cyclone changes inferred from ocean surface temperature cold wakes**

**Shuai Wang^1,*^ and Ralf Toumi^1^**

^1^Department of Physics, Imperial College London, London, UK, SW7 2AZ

^*^Corresponding author: Shuai Wang, shuai.wang@imperial.ac.uk

This document includes:

Fig. S1-S3


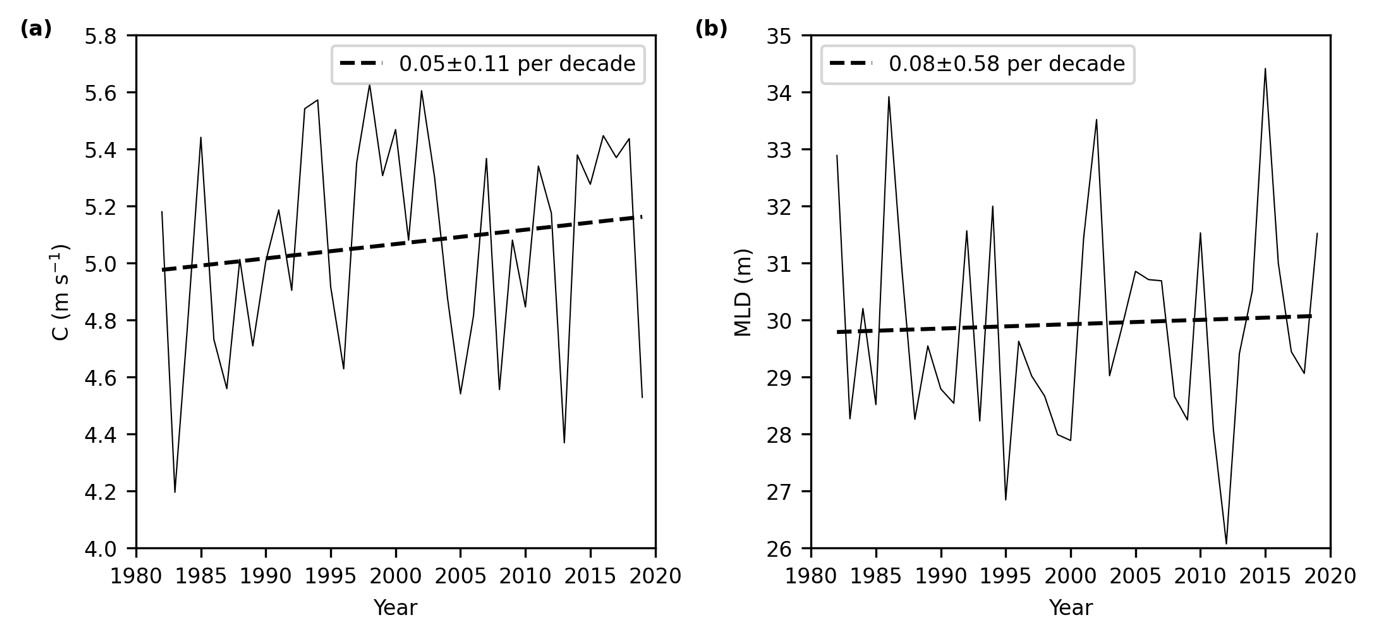


Fig. S1. Annual mean time series of global TCs. (a) 12-hr mean translation speed, C, around the time of LMI. (b) MLD. The mean±95% confidence interval of the linear trend is given in the legend.


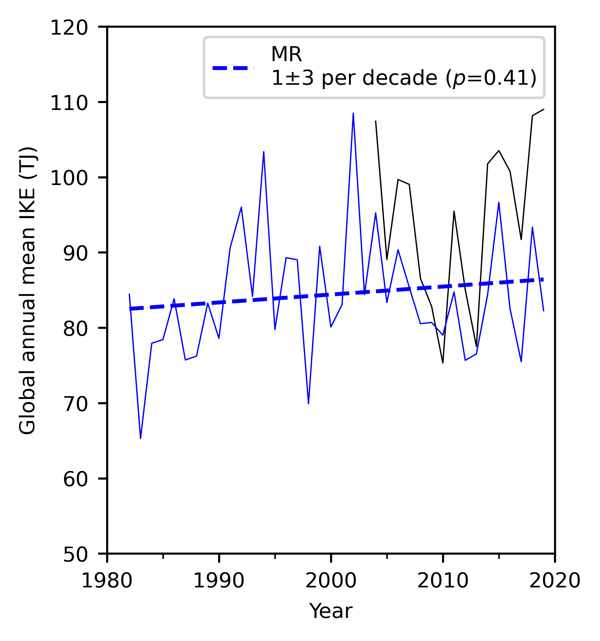


Fig. S2. As in Fig. 3e, but with Holland et al. (2010) wind profile model. For comparison, Wang and Toumi (2015) wind profile model is applied in Fig. 3e.


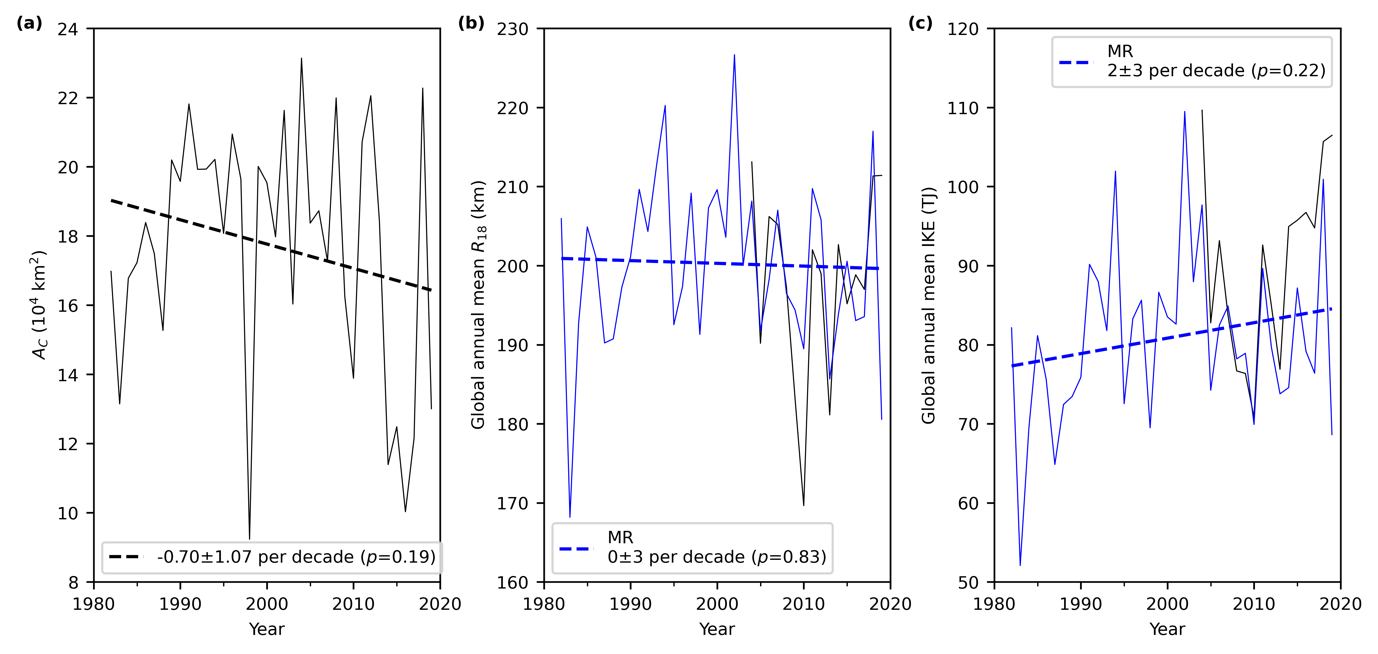


Fig. S3. As in Fig. 3b, d and e, but for TC-induced cooling area (A_C_), and (b) inferred R_18_ by using an A_C_-based multivariate regression, and (c) inferred IKE with an A_C_-based multivariate regression. For comparison, an R_C_-based regression is applied in Fig. 3.
